# Development and Prospect of Smart Materials and Structures for Aerospace Sensing Systems and Applications

**DOI:** 10.3390/s23031545

**Published:** 2023-01-31

**Authors:** Wenjie Wang, Yue Xiang, Jingfeng Yu, Long Yang

**Affiliations:** 1 School of Aerospace Engineering, Beijing Institute of Technology, Beijing 100081, China; 2 Systems Engineering Research Institute, China State Shipbuilding Corporation Limited, Beijing 100094, China

**Keywords:** smart materials, piezoelectric materials, shape memory materials, giant magnetostrictive materials, aerospace industry

## Abstract

The rapid development of the aviation industry has put forward higher and higher requirements for material properties, and the research on smart material structure has also received widespread attention. Smart materials (e.g., piezoelectric materials, shape memory materials, and giant magnetostrictive materials) have unique physical properties and excellent integration properties, and they perform well as sensors or actuators in the aviation industry, providing a solid material foundation for various intelligent applications in the aviation industry. As a popular smart material, piezoelectric materials have a large number of application research in structural health monitoring, energy harvest, vibration and noise control, damage control, and other fields. As a unique material with deformation ability, shape memory materials have their own outstanding performance in the field of shape control, low-shock release, vibration control, and impact absorption. At the same time, as a material to assist other structures, it also has important applications in the fields of sealing connection and structural self-healing. Giant magnetostrictive material is a representative advanced material, which has unique application advantages in guided wave monitoring, vibration control, energy harvest, and other directions. In addition, giant magnetostrictive materials themselves have high-resolution output, and there are many studies in the direction of high-precision actuators. Some smart materials are summarized and discussed in the above application directions, aiming at providing a reference for the initial development of follow-up related research.

## 1. Introduction

Over the past few decades, the aerospace industry has evolved rapidly. Whether in the field of civil aviation, military aviation, or space exploration, more and more requirements are put forward for the material properties used in the aviation industry. Among all the efforts to promote the development of the aviation industry, smart materials and structures are an important concern and research direction. Smart materials refer to those materials that can change their physical properties to a limited extent through certain input signal stimulation from the outside world. Piezoelectric materials, shape memory materials, and giant magnetostrictive materials are the three smart materials now undergoing the most research and application. There are also some other smart materials worth mentioning. According to the functional responsibilities of smart materials in engineering, they can be simply divided into two categories: sensors and actuators. A smart structure is a composite structure based on one or more smart materials that integrates sensors, actuators, and controllers to achieve specific functions.

As materials that can sense external conditions, smart materials can detect a wide range of signals. These signals include temperature, stress and strain, magnetic field, current and voltage, light, nuclear radiation, and so on. The properties or signals that different smart materials can change are also very diverse, including shape, stress and strain, current and voltage, damping coefficient, and so on. Different smart structures are designed based on different smart materials, with various forms and functions. AL Arsh Basheer [1] believed that the structure of smart materials should include five basic elements, namely structural materials, distributed sensors, distributed actuators, power conditioning electronics, and control strategies. For most smart structures, this generalization is somehow accurate. In the aerospace industry, the main application direction of smart material is to realize self-perception, self-diagnosis, and adaptive and self-healing functions. In this paper, we introduce and discuss the basic functions, applications, implementation principles, advantages, or disadvantages of different smart materials.

## 2. Piezoelectric Materials

**Figure 1:**
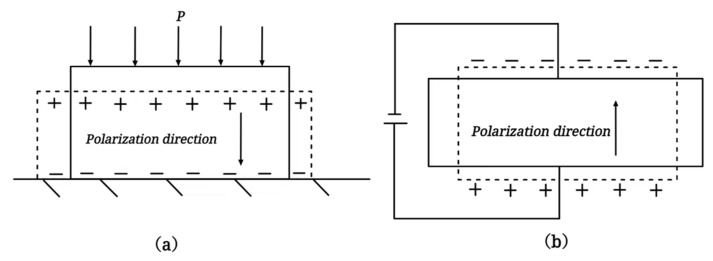
Schematic diagram of piezoelectric effect and polarization direction: (**a**) Direct piezoelectric effect; (**b**) inverse piezoelectric effect.

Piezoelectric materials refer to a class of smart materials that have a piezoelectric effect. In Figure 1, such materials can convert stress–strain signals into electrical signals, known as the direct piezoelectric effect. Similarly, such materials can also convert electrical signals into stress–strain signals, called the inverse piezoelectric effect. It is a very flexible material since the inverse piezoelectric effect enables the design of piezoelectric materials as actuators while the direct piezoelectric effect enables the usage of piezoelectric materials as sensors. Due to piezoelectric materials’ direct conversion of electrical and stress–strain signals, this material has the advantages of fast response, high-response bandwidth, high electromechanical coupling coefficient, ability to function as both a sensor and an actuator, excellent piezoelectric material integration capabilities, etc., which makes it a popular smart material. Several widely used piezoelectric materials are piezoelectric ceramics (e.g., PZT), piezoelectric polymers (e.g., PVDF), and piezoelectric composite fibers (e.g., MFC) [2]. They not only are all piezoelectric materials but also have different material characteristics and have their own advantages and disadvantages in different application directions.

Piezoelectric ceramics are the most common piezoelectric material; thus, the piezoelectric materials usually refer to piezoelectric ceramics. Compared with piezoelectric ceramics, piezoelectric polymers are a class of polymer synthetic materials; due to the difference in preparation, the mass of piezoelectric polymers is lighter, which is an important advantage in the aviation industry that pursues lightweight materials. Piezoelectric composite fiber is a new composite material designed by NASA on the basis of piezoelectric ceramics, although its essence is still piezoelectric ceramics. However, MFC has a certain degree of flexibility, in which the strain force per unit area is much greater than that of ordinary piezoelectric ceramics. In recent years, the main application research directions of various piezoelectric materials are vibration control, noise control, damage control, energy harvesting, structural health monitoring (SHM), etc.

### 2.1. Structural Health Monitoring

**Figure 2:**
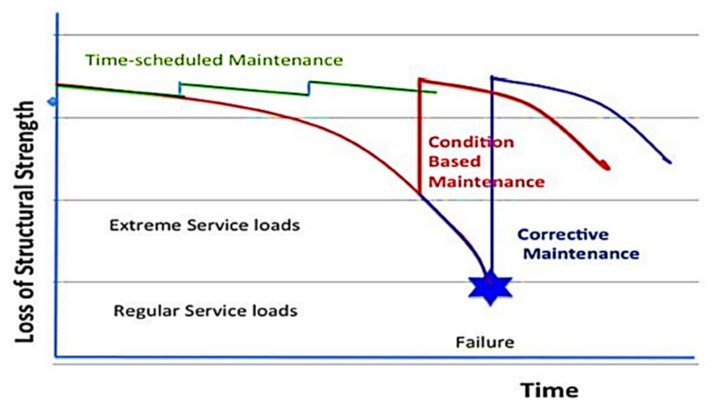
Maintenance strategies with/without SHM [3].

Structural health monitoring technology is a technology used to achieve the monitoring of the target structural health status, the purpose of which is to reduce the frequency of structural fault repair and the number of performance maintenance. Figure 2 shows the maintenance strategies with/without SHM. If combined with other repair methods, the health of the target structure can be maintained at a high level for higher safety and longer life cycle [3].

SHM technology generally monitors large structures, such as aircraft skins, so the ideal structure detector should be distributed to ensure that most areas can be covered. Moreover, the harsh environment in aerospace applications has high requirements for the robustness of sensors, so piezoelectric materials, which do not have to rely entirely on independent voltage sources or magnetic field environments but only rely on the inherent polarization of the material itself for work, have been widely used and studied in structural health monitoring technology. Mohammad Saleh Salmanpour et al. [4] studied the functionality of piezoelectric sensor-based structural health monitoring systems under different environmental conditions, which also showed that piezoelectric sensors are still reliable under harsh conditions.

Lamb wave is a kind of ultrasonic guided wave that can propagate over long distances in the structure and has sensitive behavior to damage, such as disbonding, delamination, and cracking of composite materials. As a non-destructive testing technology, Lamb waves have many advantages, and piezoelectric transducers have the function of excitation and reception of Lamb waves [5]. The piezoelectric component can be used as a generator and receiver of Lamb waves at the same time. One SHM technology implementation scheme involves distributing the piezoelectric sensor to the target structure’s predetermined detection position, obtaining the structure’s initial information by transmitting and receiving the stored ultrasonic guided wave, and determining the location and severity of the damage by comparing the difference between the ultrasonic guided wave signal and the initial signal during the subsequent structural service. In order to take preventive measures as soon as possible, an efficient SHM system must be sensitive enough to detect early matrix-related damage in addition to reflecting the structural properties following damage. Piezoelectric sensors, mechanical electrical impedance, Lamb wave technology, etc., have sensitive monitoring capabilities for small structural damage [6]. Similarly, SHM technology is also very important for the imaging ability of damaged structure and local location, which guarantees visual guidance for post-processing. The use of piezoelectric materials and Lamb wave technology through certain algorithms and processing can generate robust, high-precision imaging results, Jiaze He et al. [7]. On the basis of studying the imaging technology proposed, one should pay attention to the interference of noise on imaging technology and reduce the resulting image distortion problem.

Wang et al. [8] demonstrated that piezoelectric sensor networks can still achieve accurate monitoring of structural impact and damage areas when used in SHM technology by optimizing the wiring of piezoelectric sensor networks while significantly reducing the number of wires and monitoring channels. In the structural health monitoring of aircraft intelligent skin, piezoelectric sensor networks can realize active or passive structural health monitoring of composite structures by generating guided waves and receiving guided waves for active excitation, including damage imaging and impact imaging [9]. Hamidreza Hoshyarmanesh et al. [10] proposed a monitoring system based on a piezoelectric transducer machine electrical impedance spectrum and a portable transceiver and studied the applicability of this method, which finally confirmed the applicability of this method for early damage monitoring of rotating aerospace structures before any failure.

Although the purpose of SHM technology is to monitor the health of the structure, not all high-performance sensors are suitable for SHM, because the strength of the host structure itself is likely to be affected during embedded integration. In order to solve the problem of the influence of piezoelectric sensors embedded in the host structure, the SMART layer (Stanford Multi-Actuator-Receiver Transduction Layer) technology was proposed and studied. The SMART layer technology is a technology proposed by Stanford University to embed piezoelectric sensor elements on a flexible dielectric film through flexible printed circuits and lamination technology. Lin et al. [11] successfully used SMART layer technology to integrate distributed piezoelectric sensors on composite structures to obtain information about the condition of the structure throughout its lifetime to monitor the health of the host composite structure. Then, Qing et al. supplemented the relevant research [12,13,14] to demonstrate that SMART layer technology does not reduce the integrity of the host structure.

Based on piezoelectric materials, SMART layer technology, and ultrasonic guided wave transmission monitoring technology, Yu et al. [15] developed a real-time active intelligent diagnosis system that can realize the whole life cycle of composite structures from preparation to service, expanding the application scope of SHM technology. Deep learning has transformed traditional research methods in recent years across a growing number of areas, and it has shown tremendous promise when applied to SHM technology. [16].

Because advanced composite materials have always had a wide range and depth of applications in the aviation industry, their preparation process requires more stringent than ordinary materials. However, SHM technology has been developed to monitor the preparation process of such materials, realizing the whole life cycle monitoring from preparation to service, and improving the production quality and reliability of intelligent composite materials. Some composite materials may have issues; for instance, stress residues during the preparation process will greatly affect their service life and safety.

Konstantopoulos et al. [17] point out that since fluidity composites need to be prepared in a controlled manner, it is necessary to utilize piezoelectric materials to monitor the LCM process in order to produce high-quality composite structures. Qing et al. [18] proposed a comprehensive monitoring method based on piezoelectric sensor networks and discussed that an optimized algorithmic can be used for LCM process monitoring in addition to SHM monitoring of large structures. Liu et al. [19] used a piezoelectric sensor network to monitor the process variables of vacuum-assisted resin infusion technology (VARI), demonstrating that it is feasible to monitor the VARI process using Lamb wave and mechanical electrical impedance based on the multifunctional piezoelectric sensor network.

Using smart material sensors, such as piezoelectric materials, to fulfill the whole life cycle health monitoring of complex composite structures from production to service is of considerable significance and value, according to the findings of an increasing number of relevant studies. At present, such active real-time structural health monitoring systems throughout the life cycle are subject to the influence of piezoelectric materials themselves, and more and more in-depth verification and research of complex geometries are still needed [15]. In addition, since the placement of a large number of sensors that affect the problem of efficiency, there are also many studies focusing on the optimization of sensor patch placement (OSP) and algorithms.In terms of direct impact, the OSP can effectively reduce the number of sensors and improve overall reliability and efficiency. The OSP has a significant indirect effect on the aerospace industry since it makes it possible to monitor more complicated structures [20].

Due to their excellent Lamb wave transmission and reception capabilities, low cost, and ease of integration, as well as the use of SMART layer technology to significantly reduce the risk of compromising the integrity of the host structure, piezoelectric materials have gradually been used in the full life cycle health monitoring of composite structures from preparation to service.

### 2.2. Energy Harvesting

Energy harvesting is a relatively new application in the aviation industry. The main purpose of energy harvesting technology is to solve the energy supply problem of sensors or actuators that are not suitable for independent power supply, and the other purpose is to achieve a more cost-effective self-power supply of distributed sensor actuators, without relying on regular battery replacement or other compromising methods. In the aviation industry’s development research for the SHM technology, in order to monitor the target structure more comprehensively and stably, the sensor will be arranged in various positions of the target structure when designing. Additionally, due to the dependence of modern aviation systems on sensor reliability, as the number of sensors increases, the overall reliability of the system will however decrease. As a result, in order to address the issue of sensor failure, it is usually necessary to arrange multiple times the number of redundancies in order to minimize the effects of sensor failure on system function [21]. The energy supply problem of such a large number and widely distributed sensors requires energy harvesting technology to solve, so energy harvesting technology has also received extensive attention and research in recent years with the in-depth research and application of SHM technology [22].

The fundamental idea behind energy harvesting technologies is to convert a wide spectrum of fugitive or external energy into electrical energy through appropriate sensors and store it. An energy harvesting system usually consists of three parts: target harvesting energy, energy converter, and load. The large-scale target energy includes solar energy, wind energy, tidal energy, geothermal energy, etc., and the small-scale energy includes thermal energy, mechanical energy, electromagnetic radiation, etc. Kaur et al. [23] systematically discussed the potential of piezoelectric materials to harvest wind and mechanical energy in the environment as energy harvesting devices and claimed that this technology has important significance for wireless health monitoring technology in complex working environments. Because mechanical energy is the easiest to obtain in the aerospace industry, mechanical forced vibration or fugitive energy during active work are frequently expressed by mechanical energy, and the piezoelectric effect of piezoelectric materials can convert this mechanical energy into electrical energy, mechanical energy is usually regarded as the most efficient input energy among all the sources of energy that are currently available. Therefore, most research directions have focused on the collection of mechanical energy [24].

In order to study the collection of vibration energy by piezoelectric energy harvesters, generally attention is paid to the energy escape forms, such as galloping, vortex-excited vibration, and flutter generated by the excitation of beams, cylinders, plates, and other structures under the excitation of air flow in aerospace [25,26]. In contrast, airfoil flutter has attracted more attention from researchers, but there are a large number of nonlinear factors in the complex aviation structure, and the actual behavior often shows complex dynamic behavior, so it is necessary to conduct in-depth research and reliability verification of the relevant models to ensure the safety of the aircraft [27].

Beran et al. [28] modified the stall flutter of the airfoil section to simulate aerodynamic loads. On this basis, Bao et al. [29] used the stall flutter to excite the large vibration of the cantilever beam as an energy source and studied the influence of energy harvesting related parameters and key factors when the piezoelectric energy harvester is arranged in the appropriate position of the cantilever beam. One of the solutions for flutter-based piezoelectric energy harvesters to avoid the effects of the stall effect when simulating and studying is the introduction of different nonlinear energy sources. Zhen et al. [30] performed linear stability analysis on the basis of coupled systems, which provided theoretical support for possible flutter-based energy harvesting devices. Adam et al. [31] went a step further on the stall effect, studied the influence of pneumatic nonlinearity on the dynamic response of piezoelectric elastic energy harvesting system based on flutter, and derived the nonlinear control equation of the piezoelectric energy harvester-based system.

Research on energy harvesting technology has also been carried out on various inorganic nanomaterials, such as BaTiO_3_, PbZrTiO_3_, etc., which also show corresponding energy harvesting capabilities [32]. Compared to these materials, piezoelectric polymers (PVDFs) are a more desirable material choice for piezoelectric effect-based mechanical energy harvesting [33], so it is a common scheme to collect environmental mechanical energy by nanotechnology assembling PVDF into piezoelectric nanogenerators. In order to solve the problem of low output current density of piezoelectric nanogenerators when collecting environmental mechanical energy, Gu et al. [34] proposed a new piezoelectric nanogenerator design based on piezoelectric materials with high-voltage electric coefficient, which achieved significant improvement. Cantilever oscillators are frequently employed in industrial applications to capture vibrational energy but installing them in aerospace applications is apparently hopeless. However, it is possible to directly employ piezoelectric energy harvesters to capture vibrational energy. Tommasino Domenico et al. [35] established a coupling mathematical model of piezoelectric patch and vibration slats by modal superposition method and discussed the performance difference between cantilever energy harvesters and piezoelectric energy harvesters under ideal conditions.

A mechanical energy harvesting system is mainly composed of mechanical modules, which are responsible for receiving mechanical energy and generating electrical energy, and electrical modules, which are responsible for converting and rectifying the generated electrical energy. Therefore, the efficiency of a mechanical energy harvesting system based on piezoelectric materials depends not only on the efficiency of the piezoelectric transducer but also on how well it is integrated with the circuit. In addition to the theoretical upper limit of the target energy source, it is also necessary to consider the mechanical energy loss at harvesting, the energy loss during electromechanical conversion, and the electrical loss at output [36]. Along with concentrating on the capture of mechanical energy, wind energy is a feasible energy source in aerospace, Muhammad Abdullah Sheeraz et al. [37] pointed out that wind energy can also be used as a reliable energy harvesting source, and based on piezoelectric materials, a generalized model of piezoelectric sensing harvesting wind energy was established by first-order transmission correspondence for reliability verification.

Since the aircraft receives a large amount of air flow at high altitude, Elahi Hassan [38] analyzed the piezoelectric energy harvesters’ ability to collect energy when subjected to axial air flow using fluid–structure interaction. They also examined the pertinent piezoelectric energy harvester parameters and compared the energy harvesting effectiveness of various piezoelectric energy harvesters. In order to improve the energy harvesting efficiency under different conditions, Liu et al. [39] studied the working mechanism of the energy harvester under different wind speeds and working conditions and proposed an optimization strategy. Li et al. [40] proposed an energy harvesting model of galloping and vortex-induced vibration under geometric nonlinear factors and obtained a more accurate geometric nonlinear model of piezoelectric wind energy collector at higher wind speeds and verified it.

In general, piezoelectric materials research for energy harvesting technology has been conducted in-depth and gradually obtained research results from low degrees of freedom to high degrees of freedom and from linear to nonlinear. However, more in-depth research is still required because mechanical and wind energy exhibit complex dynamic behavior during collection. As an emerging technology, energy harvesting is part of sustainable development, and it makes sense to use piezoelectric transducers to harvest wind, mechanical, and other possible energy sources in the environment.

### 2.3. Vibration and Noise Control

Aerospace structural vibration has long been a significant safety concern, and even a small vibration can eventually result in structural incapacitation or even worse issues. For instance, the spindle structure used frequently in aerospace engineering will be affected by vibration brought on by external excitation because it performs the fundamental tasks of aircraft positioning and stabilization, and it is challenging to arrange conventional vibration control structure. However, the new wireless piezoelectric stack actuator can achieve effective vibration suppression and safeguard the safety of the aircraft [41].

Vibration control can be divided into three schemes: passive vibration control, semi-active vibration control, and active vibration control. Among them, the realization principle of passive vibration control is to convert vibration energy into internal energy or other energy forms, such as electrical energy, through the properties of the material structure itself and consume it. Active vibration control techniques include using actuators to actively modify damping or generating dynamic force, as well as distribute sensors to track the vibration of the target structure. Semi-active control is something in between. Due to the characteristics of small deformation, piezoelectric materials cannot absorb vibration energy well, so there are few studies in the direction of passive vibration control. However, piezoelectric materials have the qualities of easy integration and quick response, and because of the thorough study and application of the piezoelectric effect and inverse piezoelectric effect, piezoelectric materials can be integrated into intelligent structures as sensors and actuators at the same time. Additionally, piezoelectric materials have a high electro-mechanical coupling coefficient and the qualities of high integration without harming the integrity of the host structure.

**Figure 3:**
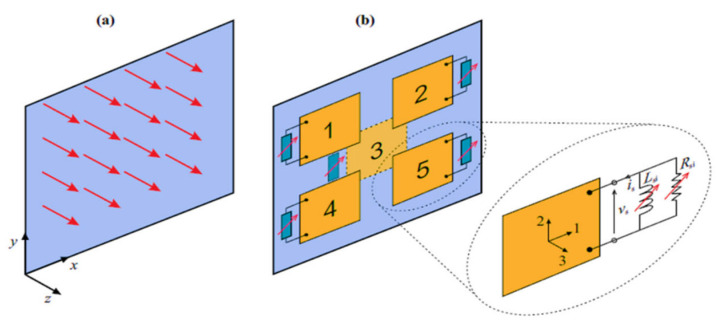
Vibration plate with shunted piezoelectric: (**a**) Stress generated by structural vibration; (**b**) Multiple piezoelectric patches integrated with microcircuits [43].

Similar to SHM technology, when piezoelectric materials are used for active vibration control, the basic implementation scheme is to distribute piezoelectric sensors into the target monitoring structure to sense the small strain of the stressed structure and realize the precise response of strain through the control logic drive controller connected to it to suppress vibration. In recent years, many different linear control methods based on piezoelectric materials have been integrated into the active vibration control of intelligent structures, and typical linear control schemes include: velocity or acceleration feedback method, optimal control method, robust control method, etc. [42] These linear control methods have some stability and robustness restrictions. Further nonlinear control method research is also being performed in order to circumvent these restrictions. Figure 3 shows a piezoelectric plate, which can sense vibration stress through multiple piezoelectric plates.

For an active vibration control system, controllability and observability are two parameters that measure its performance. In order to optimize the performance of piezoelectric materials when applied to active vibration control, Daniel Giraldo Guzmán et al. [44] applied the topology optimization principle to the structural distribution of piezoelectric sensors and actuators and combined it with the finite element method to give an active vibration control scheme with maximum controllability and observability in the feasible domain. The durability of active vibration control systems has always been important for the aircraft sector, which faces challenging environmental conditions. As a nonlinear control method, the sliding mode control method can improve the robustness of the control system better than the simple linear control method [45]. When applying the sliding mode control method for flexible aircraft control, Hu et al. [46] discovered that the superimposed modal speed feedback control method system had greater stability performance. On this basis, Jonathan Rodriguez et al. [42] applied the sliding mode control method to the active vibration control system of a piezoelectric transducer, proposed a complete active mode vibration control scheme, and verified it theoretically. Saurav Sharma et al. [47] found that this method has obvious effects on active vibration control systems based on piezoelectric sensors and actuators when performing polarization direction tuning of piezoelectric materials and proposed an active vibration control strategy based on fuzzy logic controllers through theoretical derivation and experimental verification.

MFC is a novel class of composite material that, while it possesses flexibility and piezoelectric properties, is fundamentally still made of piezoelectric ceramics. Williams et al. [48] experimentally verified the active vibration control model of cantilever beams with MFC materials. Luo et al. [49] applied MFC materials to the active vibration control of solar sail panels and proved the excellent vibration control ability of piezoelectric materials through maximum modal strain energy theory and PID control methods.

Noise control and vibration control have many similarities, such as noise control can also be divided into passive noise control, active noise control, and semi-active noise control according to the control scheme. Obviously, passive noise control is the absorption of barrier noise through the physical properties of the material or structure itself. Compared with active noise control, passive noise control materials are very mature. One implementation of active noise control is to utilize a smart foam with integrated distributed PVDF drivers to respond to the noise-induced vibrations.

By integrating piezoelectric sensors and actuators into a specific foam structure in a distributed manner, an intelligent foam structure can be created that combines passive noise control with active noise control based on piezoelectric materials. Kundu et al. [50] and Akl W.N. et al. [51] established finite element models for active vibration control and noise control, which provided a feasibility verification for finite element simulation of smart foam, and they found that, when working at low frequencies, PVDF materials deformation is small, and the noise control effect provided is not obvious. Wu et al. [52] improved the piezoelectric performance of PVDF with the electrospinning to induce higher piezoelectric properties of PVDF materials and improved the piezoelectric performance of PVDF with some structures, such as carbon nanotubes, and realized the optimization of noise control in the low and medium frequency regions. It can be expected that other fillers, such as ZnO and graphene, will be used to improve the piezoelectric response. Similarly, in order to improve the problem of the low efficiency of smart foam in the low-frequency region, Kim et al. [53] proposed to integrate the magnetic reaction material into the intelligent porous structure and realized the low-frequency efficiency improvement through a semi-active control scheme. Similarly, a sliding mode control can also be used for vibration and noise control to improve the robustness of the system, and Talebitooti et al. [54], based on a nonlinear sliding mode control method, achieved effective suppression of the vibration sound of cylindrical shells by optimizing the position of the piezoelectric actuator.

### 2.4. Damage Control

The main purpose of damage control is to prevent or delay the further destruction of damaged structures, and the other purpose is to collect as much information as possible about the damaged parts to facilitate subsequent repairs. As a type of active control, piezoelectric actuators still provide sufficient output in the damaged structure, actively changing the output force when the structure is damaged, thereby changing the mechanical properties of the damaged structure [55]. A simple implementation scheme is to directly bond the piezoelectric sensor and the actuator module to the stress concentration area. When the structure produces cracks, the force can be generated through the piezoelectric effect to reduce the stress at the cracks to achieve the effect of delaying the further destruction of the damaged structure [56,57,58]. Although this is not really a repair of the material, it can minimize damage to the mechanical properties of the structure, also known as an “active repair”.

Liu [59] proposed a two-dimensional finite element analysis model for active repair of a crack structure based on a multilayer piezoelectric patch, pointing out that the piezoelectric integrated patch should be kept at a low voltage during operation to obtain higher operational safety and economic benefits. The results showed that to achieve greater active repair performance, the piezoelectric patch’s thickness should be decreased, and its number, length, and layers should be increased. The stress intensity factor equation is a functional method that comprehensively describes crack depth, crack length, plate thickness, and plate width and can be used to predict how surface cracks will propagate under tensile or bending fatigue loads [60]. The results of a series of studies by Ahmed Abuzai et al. [61,62,63] on the stress intensity factor of the central crack produced by the piezoelectric actuator revealed that the thin piezoelectric actuator patch superimposed can perform better when utilized for active repair. In order to determine the most effective critical voltage suitable for active structural repair of piezoelectric actuators, Ritesh Kumar et al. [64] provided a general and simple solution for modeling and analyzing the repair of structural cracks by analyzing the repair performance of piezoelectric materials under thermal-mechanical loads, and gave a reference effective critical voltage.

The delamination of composite structures is also a common damage, and Sohn H et al. [65] proposed a composite layering monitoring method based on piezoelectric sensors, which actively generates specific waveforms through piezoelectric patches for response signal monitoring and realizes the identification of delamination phenomena by separating damage characteristics from the original signal. Hameed M. Saqib et al. [66] optimized the method and proposed to use Lamb wave-based time compensation signals to achieve better imaging of damage. Hong et al. [67] proposed, by applying the nonlinear detection method to the damage detection system of piezoelectric materials, that nonlinear higher-order harmonics are very sensitive to layered damage, and piezoelectric sensors can detect damage to pipeline structures by separating this signal. Flutter is very common in aerospace applications, but it can be a fatal problem for damaged structures, Kuriakose et al. [68] simulated structural damage by destroying fibers during composite manufacturing, combined with pneumatic testing of flutter characteristics before and after structural damage, and using piezoelectric patches to improve the stress conditions of damaged structures, achieving the goal of limiting the flutter boundary of damaged structures to a safe area.

## 3. Shape Memory Materials

**Figure 4:**
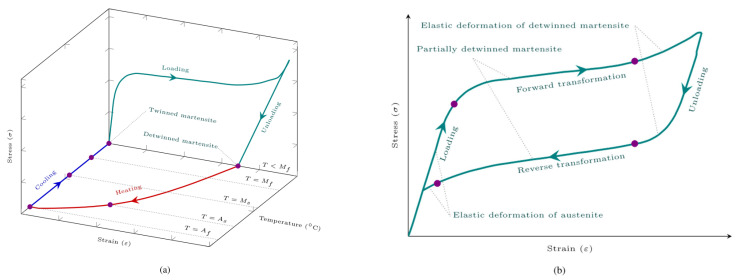
Stress–strain–temperature curves for (**a**) shape memory effect and (**b**) super-elasticity [69].

Shape memory material is a kind of smart material with unique shape memory effect and super elastic effect because it can respond to changes in temperature, pressure, and other changes to produce recoverable deformation, so it is considered to have a similar role to “memory”, mainly divided into shape memory alloys and shape memory polymers. In Figure 4, stress–strain–temperature curves for shape memory effect and super-elasticity can be found. SMA has the advantages of a large driving force per unit volume and a direct actuation of the material itself, thereby reducing system complexity and improving system reliability and low-motion noise when the SMA mechanism actuating. The requirements for smaller size, lighter weight, and robustness pursued by the aerospace field make smart materials, such as SMA, widely used in aerospace.

SMA material is a material that is mainly affected by temperature and stress and achieves solid–solid phase conversion in the two states of martensite and austenite, and the parent phase of martensite and austenite is the shape that such materials can “remember”. Austenite is a state in which metals are characterized by cubic crystal structures that are stable at relatively high temperatures. Martensite is a state in which metals are characterized by monoclinic or tetragonal crystal structures that are stable at relatively low temperatures or under high stress.

The one-way memory effect refers to the deformation of SMA at lower temperatures, i.e., in the martensitic state, and when the temperature rises to the austenitic state, the metal returns to its pre-deformation shape. The two-way memory effect refers to the fact that the metal returns to the austenitic phase at a higher temperature austenitic state and returns to the martensite phase when cooled to the martensite state. In addition, the all-round way memory effect is more unique; it not only has a two-way shape memory effect but also in the process of temperature change. Always following the same shape change law, it can be said that it not only remembers the shape of austenitic phase and martensitic phase but also remembers the intermediate process of deformation. This spontaneous shape change in the process of temperature cycle change is greater than all other reversible shape memory effects, and the shape of the high temperature phase and the shape of the low temperature phase are completely opposite memory effects called the all-round way memory effect.

In general, the shape memory effect means that when SMA is in a low-temperature twin martensite structure, it is transformed into a non-twin martensite by loading, and when the temperature rises, the SMA will be converted to austenite and produce corresponding deformation. Current shape memory polymers (SMPs) can also produce shape memory effects based on changes in magnetic flux, current, or pressure, and the principle is similar.

Super-elasticity refers to the characteristic that SMA converts austenite into non-twin martensite and deforms by loading and then quickly returns to austenite and returns to its initial shape after unloading. It is characterized by the fact that it does not produce permanent deformation even under large loads and is an important characteristic that SMA material structures can replace springs and other structures. The stiffness of shape memory polymers and shape memory alloys are very different in temperature, and the elastic modulus and yield stress of shape memory alloys are higher at high temperatures and lower at low temperatures, but the opposite in shape memory polymers [70].

In the aerospace industry, using the shape memory effect, SMA can be made into an excellent intelligent structure integrating drive and structure, thereby greatly improving system reliability. Super-elasticity, by contrast, allows structures made of SMA materials to withstand enormous strains and still return to their initial shape, which is very suitable for some applications where vibration energy absorption or extreme deformation recovery is restored. SMP can be divided into thermos-induced SMP, electro-induced SMP, photoinduced SMP, chemically induced SMP, etc., according to its recovery principle. For example, the function of thermos-induced SMP shape memory mainly comes from the existence of incompletely compatible two phases inside the material; that is, the stationary phase that maintains the shape of the molded product and the reversible phase will soften with temperature change and harden with reversible change. The function of the stationary phase lies in the memory and recovery of the original shape, and the reversible phase ensures that the molded product can change shape. Electro-induced SMP is a composite material of thermoforming shape memory polymer materials and conductive substances (such as conductive carbon black, metal powder, and conductive polymer). The memory mechanism is the same as that of thermos-induced shape memory polymer, and the composite material increases the temperature of the system through the heat generated by the current, resulting in shape recovery, so it has both conductive properties and good shape memory function.

### 3.1. Shape Control

Shape control is one of the main application directions of shape memory materials in the aviation industry. The main purpose of shape control is to adapt to different environments by changing the shape of structures, such as aircraft skins. Generally speaking, high-speed aircraft do not perform well at low speeds, and the same low-speed aircraft cannot work stably at high speeds. The reason is not only related to the power system but also related to the shape of the aerodynamic control surface, so there is hope to reconcile this contradiction through the controllable deformation characteristics of shape memory alloys. In addition, the unfolding or retracting of structures, such as a spacecraft panel, is also a shape change that can be controlled by shape memory alloys.

Shape control technologies, though they are all controlled by shape memory alloys, can be roughly classified into two categories based on various implementation methods. One is indirect shape control, the implementation principle of which is to use shape memory alloys to replace traditional mechanical or hydraulic actuation and other mechanisms to achieve shape control. Due to its own strength and actuation ability, shape memory alloys can greatly simplify the structural design and reduce the complexity when replacing traditional actuated structural parts, thereby improving the reliability and stability of the structure, and making it easier to repair and replace.

**Figure 5:**
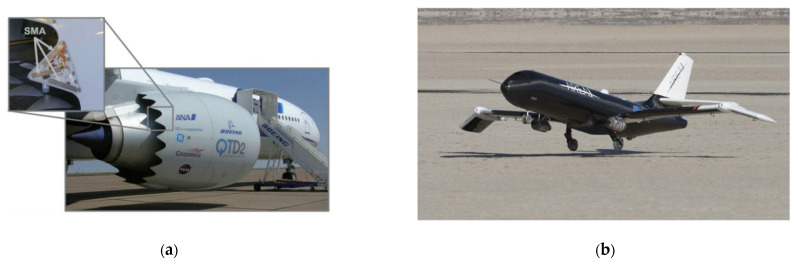
Examples of SMA materials in the aviation industry: (**a**) SMA-based shape control at Boeing 777-300ER airplane engines [78]; (**b**) SMA torque tubes are mounted in the chord-wise direction along the hinge-line to control the articulation of the outboard wing section.

The SAMPSON Smart Air Intake is a project that uses shape-memory alloy actuators to replace traditional hydraulic actuators, adjusting the geometry of the air intakes through SMA actuators to assisted subsonic attack aircraft in successfully performing supersonic interception missions in this way [71,72]. Similarly, an RRB project was developed to integrate SMA actuators into the rotor blade construction of the aircraft. This project utilizes SMA actuators to optimize the traditional structural configuration and significantly enhance performance [73]. The results of the RRB project showed that the small protruding structure on the rotor blades can effectively reduce the noise caused by the blade–vortex interaction, but at the same time, it will also reduce the performance of the overall structure due to increased resistance [74]. In order to further reconcile this contradiction, Boeing developed a rotor blade structure based on SMA actuators, which uses SMA actuators to expand the structure to reduce drag when the rotor blades need to be quiet and to retract the structure when they are not needed to reduce noise [75]. Turbofan engine noise is a common problem for large jets, and zigzag or V-shaped jet port devices [76] have been shown to significantly reduce vehicle noise, but the problem is that such structures can impair the aerodynamics of the engine. The VGC project has achieved great success in this direction through SMA actuators to transform the local structure of the aircraft between the jagged noise-reducing shape and the shape during cruising while taking into account the advantages of both [77]. The common feature or exploration direction of the application of these shape memory alloys is the use of shape memory alloys to reconcile the contradiction between the shape requirements of different functions in aircraft design and also provide a direction for subsequent research and application. Figure 5 shows some examples of SMA materials in the aviation industry.

The general deformation structure itself requires the necessary control structure, which will lead to additional problems, such as surface discontinuities and being overweight [79]. Another idea is direct shape control; the implementation principle is to cancel the traditional actuated structure, and the shape memory alloy is directly integrated into the deformed target structure, such as skin, through the shape memory effect to directly achieve deformation. The all-round way memory effect of shape memory alloy makes it not only able to remember two different shapes but also to remember the deformation process in the middle, making it more controllable in the process of deformation. Ideally, as long as the appropriate parent phase shape is preset, it can be directly deformed by the distributed memory alloy drive. Since there is no traditional mechanical or hydraulic actuation mechanism, the deformation structure is a whole, which can improve the reliability of the system. Compared with indirect shape control, there are fewer research results on direct shape control, especially the study of adaptive skinning, most of which are still focused on the low subsonic speed of UAVs, and although the failure of the deformed wing will have serious consequences, the corresponding failure research is still less [80].

Shock waves present a challenge for transonic wings, increasing the drag as aircraft work, and one solution to obtain better aerodynamic performance is to form a controlled bulge structure on the wing through SMA to reduce the drag caused by shock waves [81]. In order to train shape memory alloys with suitable properties, Qiu et al. [82] proposed a thermomechanical training program and designed a complete device for training two-way memory effects. Compared with traditional spoiler actuators or pressurized elements, shape memory alloy actuators have the advantages of small size and high controllability [83]. Balzarek et al. [84] attempted to integrate SMA actuators into the helicopter’s wings to form an elastic skin for better hover flight capabilities. On this basis, Ameduri et al. [85] systematically studied the influence of different parameters on the adaptive deformation ability of rotating wings and established a database that can be used to predict model behavior in subsequent related studies. In addition to this, Courchesne et al. [86] studied the aeroelastic characteristics of applying SMA directly to the skin of deformed wings. In order to obtain better adaptive deformation wing structural materials, Ashir et al. [87] integrated SMA filaments into fiber-reinforced plastics, tested the deformation behavior of adaptive deformed wings, and showed that SMA can achieve the highest deformation under the current intensity of 1A and the heat induction conditions of 60s. In order to simplify the structure of controlling the flexible skin using SMA brakes, Hajarian et al. [88] proposed that the SMA line be integrated into the structure through a silicone adhesive to obtain an adaptive deformation airfoil result that can be continuously deformed, and finally, the intelligent airfoil was simulated and mechanically tested.

### 3.2. Low-Shock Release

Low-shock release is a technology used to separate or deform structures in space, with the aim of solving the problem of impact on systems caused by traditional explosive separation release devices and the resulting mission failure [89]. In the low-shock release device, shape memory materials play a key role, because shape memory materials are smooth and quiet in the process of deformation, and shape memory materials can be driven by continuous and controllable temperature changes, which is more reliable and safer under complex aerospace application conditions. The low-shock release technology based on shape memory alloy has apparent advantages over the traditional explosive release mechanism, such as being pollution-free and reusable. It is better suited for the connection and separation of precise instruments and equipment due to its silent and smooth working manner.

In order to complete different missions, small satellites will perform various systems’ pull-out, separation, and other work more frequently, and low-shock release devices based on shape memory alloys are ideal for their design [79]. The simple principle of single-stage release mechanism is to directly drive the displacement of the connecting device after the SMA is heated so as to lift the limit position of the load-bearing structure and complete the task of low-shock release. The single-stage SMA separation actuator developed by Yan et al. [90] has a maximum preload of 40 kN and uses two redundant SMA wires to improve the reliability of the system, and the life test shows that it can automatically reset the work for more than 50 cycles and is robust to typical thermal and vibration loads. The short cycle life of the single-stage release mechanism is a drawback, and the multi-stage release mechanism, which has a longer service life, is better suited for spacecraft that must conduct release duties repeatedly. Huang et al. [91] believe that an important factor affecting the service life of the SMA release mechanism is due to the excessive trigger force, and on the basis of limiting the maximum preload to 15 kN, an SMA release device using a two-stage ball lock is proposed, which ultimately makes the cycle life exceed 693 times. Yang et al. [92] designed a release device based on SMA fiber and carried out comprehensive performance tests, which have a higher response speed than the traditional SMA release device. At present, a variety of SMA release devices have been applied in orbit, replacing the traditional pyrotechnical explosion separation device to obtain a safer separation and release function. SMA release devices are also gradually becoming lighter and more automated in order to provide more stable functions while reducing mass and footprint.

Shape memory polymers (SMPs) also offer unique advantages in low-shock release applications, where SMP-based integrated components are lightweight, reliable, and economical. Li et al. [93] realized the geostationary orbit in-orbit space test of SMP and obtained many useful test data. On this basis, in order to meet the low-shock release requirements of the release device by the deployment of solar sails of satellites in orbit, Xin et al. [94,95] designed a smart solar array based on shape memory materials, which is locked by one SMA and two SMP release mechanisms, with a maximum preload of 2000 N, so it is suitable for the limit and release of separable structures with small mass. Without the assistance of a conventional motor controller, the SMP release mechanism achieves almost 100% shape recovery after heating in the 60 s and can be reused.

### 3.3. Vibration Control and Impact Absorb

Shape memory materials have a different elastic modulus and stiffness coefficient at different temperatures, and the elastic modulus and yield stress of shape memory alloys are higher at high temperatures and lower at low temperatures but the opposite in shape memory polymers. Using this property, after the shape memory alloy fiber is integrated into the composite structure, as long as the active or passive temperature changes, the stiffness coefficient and natural frequency of the structure can be changed to achieve the purpose of an indirect control of vibration. For example, in the interior or surrounding environmental structure of the engine, there are many tubular structures; these tubular structures are subjected to thermal loads when the engine is working. Ju Qiu and Ion Stiharu [96] tried to integrate SMA fibers into aluminum tubes and composite layers so that when the temperature rises, its stiffness and the natural frequency of the structure change so that the structure can be avoided under the excitation frequency and effectively suppress the occurrence of resonance phenomenon.

As long as the SMA material is thermally trained in advance, vibration suppression can be achieved by embedding it in the composite structure [97]. Garafolo et al. [98] tested the active vibration control ability of the silicone plate structure embedded in the SMA wire under the aerodynamic flutter generated by the subsonic wind tunnel and successfully reduced the bending amplitude of the structure by more than 30%. Mani et al. [99] integrated SMA into rotating blades and provided an analytical model for driving intelligent blades, according to which showed that the SMA-embedded structure can minimize the maximum vibration displacement by 47% in comparison to ordinary rotating blade constructions. Kuo et al. [100] utilized the finite element method to numerically study the inhibitory effect of SMA in the flutter effect, and the results showed that the control effect of smart board structure on flutter could be effectively strengthened by increasing the volume fraction of embedded SMA fibers. On this basis, Lin et al. [101] provided an analytical model of nonlinear aeroelasticity, flutter delay, and random response of SMA composite plate in a therma–pneumatic–acoustic coupling field. They compared the recovery stress and flutter pressure of SMA to verify the reliability of the model, and the results demonstrated that embedded SMA can not only significantly improve the vibration characteristics of the composite plate but also accelerate the stability speed of the panel, which provides a vibration solution for the design of aircraft. Danish et al. [102] further studied the 3D SMA composite, conducted wind tunnel experiments on it under subsonic conditions, and evaluated the vibration control ability of the three-dimensional structure after embedding the SMA wire. The results showed that the embedding SMA helped to lower the flutter velocity and frequency.

In addition to controlling the stiffness of composite structures, such as continuous shape memory alloy strips, there is another vibration control scheme. Shape memory alloys have the critical property of super-elasticity, which prevents permanent deformation even when subjected to large loads and allows for speedy recovery after unloading. These materials have a large hysteresis loop area and can produce high intrinsic material damping [103]. A vibration control scheme based on super-elastic shape memory alloys passively consumes vibration energy through shape memory alloys, while ensuring that the structure can still recover sufficient stiffness when needed. Saadat et al. [104] systematically discussed the limitations of shape memory alloy super-elasticity in vibration control.

In order to isolate the vibration problem brought on by the reaction force of the transmitting device in space, Kwon et al. [105,106] proposed a mesh washer for the passive isolation of vibration. The effectiveness of the structure is demonstrated by the basic characteristic test of the SMA mesh gasket and the vibration test of the emission environment under orbital conditions. This mesh isolator can successfully minimize the shock load of the structure during the flight phase of launch vehicles and satellites, but it will amplify the low-frequency vibration generated by engine thrust and aerodynamic load. In order to solve this problem, Qiu et al. [107] proposed that the method of frequency tuning can be used to suppress the low-frequency amplification of SMA vibration isolation washers.

It is also feasible to enhance the impact performance of super-elastic SMA materials by embedding them in composite structures, utilizing the super-elasticity of SMA materials in a manner akin to active vibration control. Wei et al. [108] confirmed that this method can effectively improve the impact performance of the structure by embedding SMA materials into composite structures. Then, Meo et al. [109] utilized the finite element simulation method to conduct low-speed impact tests on composite materials embedded with super-elastic SMA wires. They discovered that, under all test conditions, SMA-embedded composite structures demonstrated better damage resistance to low-speed impact than traditional composite materials. This is because SMA materials have special super-elastic and hysteresis characteristics that can absorb mechanical energy on impact. As a polymer material with excellent integration performance, fiber-reinforced plastic can also effectively improve its impact load resistance by embedding SMA material. Amit et al. [110] studied the influence of the position and quantity of SMA material embedding on the structural properties and gave the best position and quantity reference opinion for SMA wire arrangement.

In contrast to conventional elastic materials, shape memory alloys can endure large loads without easily deforming permanently and can swiftly revert to their original shape after unloading owing to their super-elasticity. In order to realize the adaptive function of the structure to external stress, NASA utilized a shape memory alloy to develop a Mars rover tire that is woven from super-elastic shape memory alloy wire, which can not only achieve adaptive ability to road surface unevenness input during mission execution but also overcome the plastic deformation problem of traditional spring tires [111].

### 3.4. Connector and Seal

In the aviation industry, under the internal and external excitation, the pipeline system will produce structural vibration, and the fluid in the pipeline will also produce pressure pulsation. These vibrations will be coupled with each other to form complex waves propagating within the structure. During the development of the aircraft, such fluid–structure interaction will cause pipeline fatigue failure, oil leakage of accessories, unstable structural connection, and other problems [96]. In addition to vibration control, there is another way to improve structural reliability, that is, to utilize the shape memory alloy driving force and the characteristics of shape memory. The SMA tube first expands the inner diameter at low temperature to connect the structure, and then heats it so that the shape memory alloy produces a restoring force and deformation, which presses the sealed structure to be connected with excellent sealing performance. SMA fittings are highly reliable, simple to install, do not require welding, and are frequently used in a variety of industrial fields [112].

As early as the 70s of the 20th century, the US military already applied the technology of SMA pipe joint sealing on its F14 fighters [113]. In order to solve the problem of a poor shape memory effect of general Fe-based SMA, Jee et al. [114] proposed a scheme of using both SMA coupling and auxiliary coupling inserted into the pipe to obtain better sealing performance. Cao et al. [115] tested the bending fracture strength of three common SMA pipe joint expansion methods, namely radial expansion method, unidirectional pressing method, and bidirectional pressing method, and found that the bending fracture strength of a SMA pipe joint could be significantly improved by changing the appropriate diameter expansion method.

### 3.5. Assist Self-Healing

Shape memory materials also have important applications in the assisted self-healing of material structures. The development of SHM technology has enabled the goal of self-monitoring of smart material structures to be achieved, but the realization of self-healing functions of material structures is still a more attractive goal. Self-healing materials can be roughly divided into endogenous self-healing materials and exogenous self-healing materials according to the classification method of repair sources. Exogenous self-healing materials, such as microcapsules and vascular network self-healing materials, refer to the preservation of raw materials for repairing structures outside the material by certain methods and only combined with the material when needed. Endogenous self-healing materials refer to the use of the properties of the material itself to achieve self-repair and connection at the molecular level, such as thermoplastic and thermosetting materials [116].

**Figure 6:**
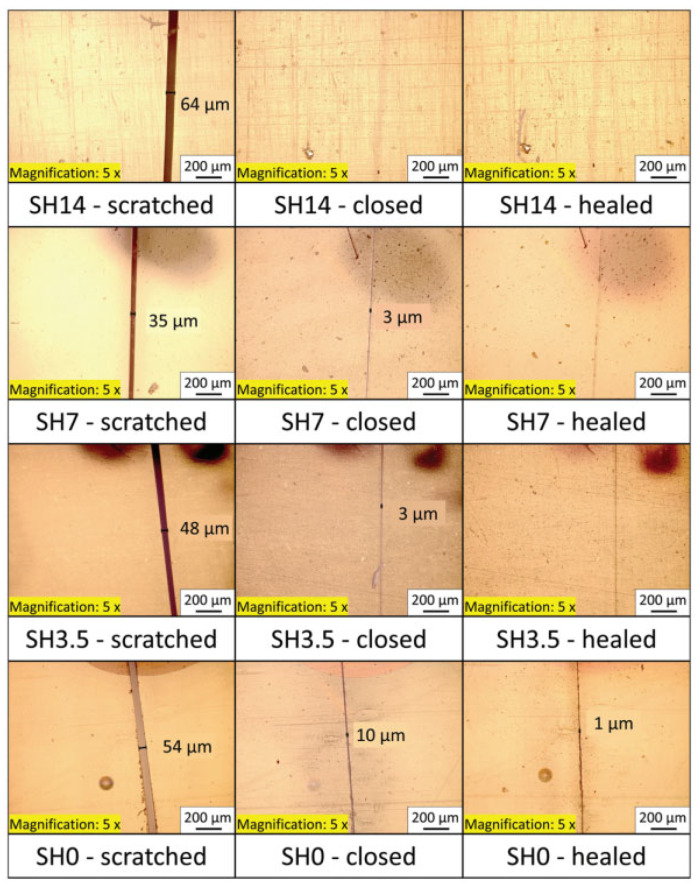
Morphological investigation of the SMASH performance of the selected networks via optical microscopy. The samples were monitored as-scratched, recovered (closed), and after healing at 180 °C for 5 h [117].

Regardless of whether it is an endogenous or an exogenous self-healing material, the damaged structure must fit adequately at the physical level because the material itself cannot have strong fluidity. However, some materials have excellent fluidity under certain conditions, but it is still challenging to self-stitch. Therefore, it is noted that shape memory polymers can not only remember the initial state of the structure but also adapt to its deformation. In Figure 6, the shape memory effect can be activated by simply applying signals during repair, such as temperature, magnetic field, current, etc. This allows the damaged structure to first attain the physical macroscopic level of fit before relying on the inherent qualities of the material to cure itself.

Kuang and Cantwell [118] studied composite materials with integrated SMA and concluded that it is feasible to limit crack propagation with the shrinkage force generated by continuous SMA. Subsequently, Saeedi et al. [119] tried to use SMA wires to control the fracture behavior of composite structures, and the experimental results showed that a 67% increase in crack propagation resistance was observed at 1% pre-strain SMA. The successful application of damage control shows that SMA can effectively provide an active closing force to limit crack propagation, and at the same time, the microcapsule self-healing material alone requires that the crack of structural damage should not be too large. So, Kirkby et al. [120] tested the auxiliary effect of integrating SMA into the microcapsule self-healing material. The results demonstrated that the SMA wire can be activated to close the crack during healing when the crack propagation ruptures the microcapsule to release the repair solution. Even with a huge crack, activating the SMA wire first can greatly enhance healing performance. Poormir et al. [121] studied the healing capacity of SMA-assisted self-healing materials using the Taguchi method at various pre-strain and activation temperatures. The results showed that the application of a healing temperature of 190 °C under the condition of 6% pre-strain can maximize the ultimate tensile strength. Thermoreversible polymers are another smart material that can achieve self-healing in addition to auxiliary microcapsule self-healing materials. Saeedi et al. [122] utilized thermoreversible polymers as a matrix, along with the integrated SMA reinforcement material to develop a new self-healing composite material. This structure can activate SMA by heating to provide auxiliary force, while activating the chemical reaction of the ther-moreversible material itself to realize self-repairing. This actively enhanced structure has an average healing efficiency of 92%.

Fiber-reinforced polymers are widely used in aircraft, and the impact of aircraft may cause invisible damage to the fiber-reinforced material structure. Jony et al. [123] studied the healing efficiency of CFRP by adding shape memory polymer and polycaprolactone (PCL) healing agent to carbon fiber-reinforced composite (CFRP) and suggested that this SMP-assisted self-healing material could be applied in aerospace structures. On this basis, Konlan et al. [124] developed a self-healing composite laminate with improved transverse strength and circulatory healing ability to solve the possible delamination problem under low-speed impact. Studies revealed that the structural stratification after SMA assistance was significantly reduced, and even after several shock-healing cycles, it still showed excellent layering healing ability.

Achieving structural self-healing has long been a desirable aim for the aerospace industry, which strives for structural reliability, but there are currently few real application examples based on the present state of research on shape memory-assisted self-healing materials.

## 4. Giant Magnetostrictive Materials

**Figure 7:**
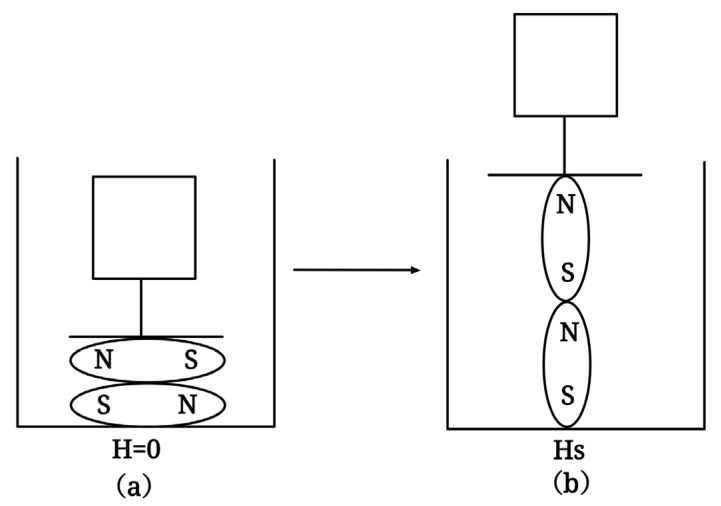
Two-dimensional schematic diagram of magnetostrictive effect: (**a**) No applied magnetic field; (**b**) an external magnetic field is applied.

Magnetostrictive materials are a unique smart material, which was originally limited by the limitations of magnetostrictive materials and did not have the same continuous development as other smart materials. The giant magnetostrictive effect, however, has been discovered and applied since the application and research of rare earth elements, and the magnetostrictive effect has now reached a sufficient application level at room temperature. Compared with piezoelectric materials, magnetostrictive materials have a higher energy density and an excellent inherent robustness, so magnetostrictive materials have considerable research and application value [125]. Its disadvantages are also obvious; that is, the hysteresis and complex physical properties of magnetostrictive materials restrict its design and application. Magnetostrictive materials can be approximated by a set of magnetic domains, the orientation of which depends on the interaction of magnetic and mechanical energy [126]. In simple terms, magnetostrictive materials exhibit a property that produces stress–strain under a magnetic field. In Figure 7, when no magnetic field is applied, the orientation of the magnetic domain is random, and the overall state is in a low-energy state and is more stable. When an external magnetic field is applied, the orientation of the magnetic domain tends to be the same, and the whole shows a change in displacement, which is called magnetostrictive.

Terfenol-D and the Fe–Ga alloy Galfenol are the two magnetostrictive materials that are now getting most research. Terfenol-D is a metal of rare earth elements with obvious magnetostrictive effects. Galfenol is a Fe–Ga alloy material with a weaker magnetostrictive effect than Terfenol-D, which can reach saturation at lower magnetic field strength and has high permeability and good mechanical properties [127].

### 4.1. Guided Wave Monitoring

Ultrasonic guided wave detection technology is a non-destructive testing technology, compared with traditional magnetic particle detection, radiographic inspection, and other non-destructive testing technology. It offers considerable advantages in large-scale long-distance structural damage monitoring in the aviation industry because of its low frequency of usage, long propagation distance, small signal attenuation, etc. In addition to the piezoelectric guided wave sensors mentioned above, magnetostrictive materials have good sensitivity and service life when applied to electromagnetic guided wave sensors. The guided waves excited by piezoelectric sensors are typically multimode and dispersive, and additional design considerations are required to obtain higher performance guided wave signals [128]. In terms of guided wave excitation ability, magnetostrictive patch sensors (MPT) based on electromagnetic coupling mechanism can more easily obtain single-mode, non-dispersive guided waves [129], which makes magnetostrictive materials promising to replace piezoelectric materials as smaller sensors for structural health monitoring with higher performance.

Zhou et al. [130] studied the use of magnetostrictive sensors to generate a non-dispersive shear wave SH0 to monitor the damage of non-ferromagnetic plate structures, and experimentally demonstrated the effectiveness of magnetostrictive sensors after optimizing the design with the commercial software, ANSYS. The MPT, developed by Park et al. [131] can be utilized for non-destructive testing of thin-walled structures. It is a compact omnidirectional shear horizontal (SH) wave MPT that only needs two annular plaques to generate a specific SH1 mode detecting wave. Although both have long-range non-structural testing capabilities, the practice of structural health monitoring using guided waves relies more on Lamb waves than SH waves since Lamb waves are simpler to produce in actual engineering structures [132]. Subsequently, Lee et al. [133] proposed a MPT with an axisymmetric configuration to generate omnidirectional Lamb waves and studied the generation mechanism and frequency characteristics of Lamb waves. In order to overcome such problems, Xie et al. [134] proposed a new MPT consisting of alloy patches and double-layer flexible printed circuits, which can be used for health monitoring of thin-walled structures with curved surfaces. Sha et al. [132] established a multiphysics finite element model of on-board magnetostrictive transducers and studied the design variables and structures required for magnetostrictive transducers for structural health monitoring.

### 4.2. Vibration Control and Energy Harvest

When magnetostrictive materials are used for vibration control, they are also divided into three types of directions: active vibration control, passive vibration control, and semi-active vibration control, which are generally used for the suppression of small amplitude and high-frequency vibration. Magnetostrictive materials have a higher loss factor than piezoelectric materials. Passive control mainly uses the hysteresis effect and eddy current of ferromagnetic materials to dissipate vibration energy. Magnetostrictive materials have higher stiffness than other materials, making them ideal for structural damping applications when high stiffness is required. Of course, by combining other smart materials, composite materials can be prepared to overcome these shortcomings.

**Figure 8:**
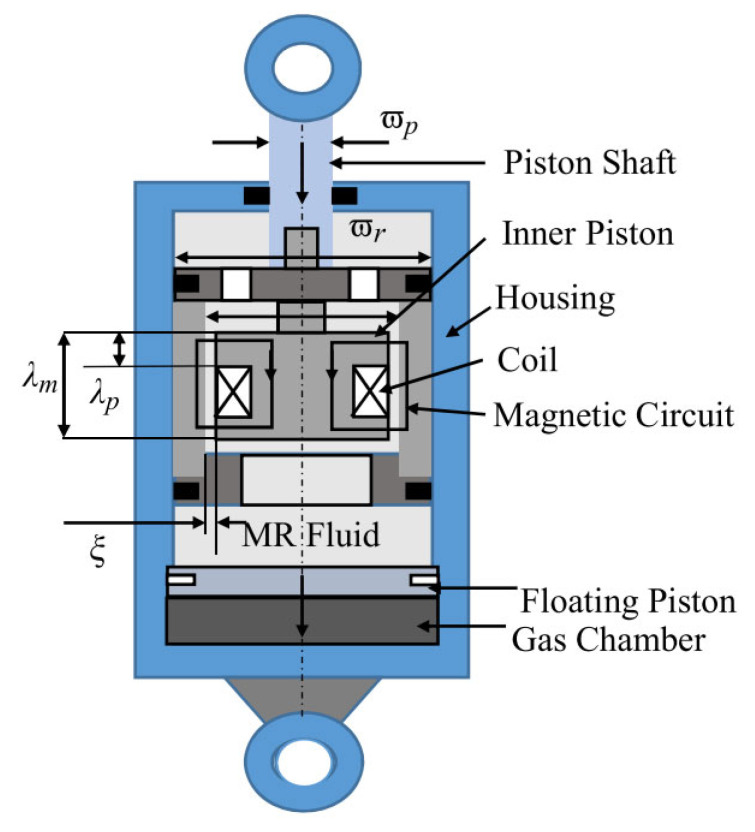
Schematic configuration of the MR damper [140].

When magnetostrictive materials are used for active vibration control, in addition to the traditional control scheme, that is, the use of dynamic forces or deformation to suppress vibration. Zenkour et al. [135] synthesized various theories applied to magnetostrictive composite plates, conducted theoretical analysis and numerical tests on the vibration control capability of smart board structures embedded in magnetostrictive layers, which provided a theoretical analysis foundation for related research. Vibration energy can also be transformed into electrical energy due to the Villari effect of giant magnetostrictive materials [136]. Yan et al. [137] proposed a new vibration energy harvester based on Terfenol-D and tested the actual prototype. It was confirmed that this new vibration energy harvester can produce significantly higher effects than traditional vibration energy collectors and can withstand the impact of 20–30 Mpa, which has a collection and suppression effect on the long- and short-term vibration excitation in the aerospace industry. Magnetorheological material is a magnetostrictive material that disperses ferromagnetic particles in a base carrier or elastomer, which can obtain controllable stiffness and a damping coefficient through a variable magnetic field applied by the outside world. Based on this property, vibrations can also be suppressed, if necessary, by changing the stiffness and damping coefficient of the structure. Magnetorheological materials are used in the vibration control of aerospace precision platforms [138], landing gear [139], and other structures. Figure 8 shows a MR damper, which is utilized to vibration control.

In order to use the giant magnetostrictive material for the whole spacecraft vibration control technology, Xu et al. [141] constructed a vibration control and energy harvesting device for the entire spacecraft system by integrating the giant magnetostrictive material into the nonlinear energy sink (NES) and then embedded the NES-GMM structure into the scale model of the entire spacecraft system for experiments. The vibration control and energy harvesting under working conditions and parameters have improved effects. The peak value may be efficiently lowered at the resonant frequency, and the maximum voltage collected by the device at the formant peak can reach 4 V. The outer surface of aerospace structures is usually subjected to frequent temperature changes, such as space structures alternating between sunny and shady surfaces, and thermally induced vibration has a dangerous impact on the safety of such structures. Mirzavand et al. [142] proposed an active thermally induced vibration control scheme embedded in the plate structure through the GMM telescopic layer, and the appropriate deformation can be generated by the GMM telescopic layer to suppress the vibration.

### 4.3. High-Precision Actuators

The response speed of giant magnetostrictive materials, such as Terfenol-D and Galfenol, which are representative of the smart material actuator, is faster than that of general materials. This new type of actuation mode has the advantages of simple structure, high-frequency drive, and high-resolution displacement output accuracy. It is an important direction for the development of actuator components for future aircraft [143]. Giant magnetostrictive materials are not easily aging compared to piezoelectric materials with drift, aging, overvoltage breakdown, and other issues. Even if their magnetostrictive effect briefly disappears after reaching the Curie temperature, it returns to normal after returning below the Curie temperature, and there will be no thermal failure issue. This has a high degree of safety and reliability under complex aerospace applications.

A high-precision vibration suppression platform was proposed by Wang et al. [144] that combines a magnetostrictive actuator with a passive vibration absorption device. The device utilized giant magnetostrictive material as a dual-output actuator to ensure that each output point moves consistently. The displacement output of a sufficient application can then be produced through the displacement amplifier. Sun et al. [145] designed and studied an integrated actuator that can solve the problem of precise positioning and vibration control of spacecraft optical components at the same time, and the experimental results showed that this integrated actuator had excellent vibration suppression ability and high-precision output performance in low-frequency bands. Recent studies have shown the feasibility of GMM materials as self-sensing actuators, and it is believed that GMM actuators have the potential to realize the functions of self-sensing and precise positioning at the same time, which can further simplify the structure of the system [146,147]. On this basis, Xie et al. [148] developed a new self-sensing actuator made by utilizing the variation in GMM Young’s modulus under various external situations. The results showed that this new actuator had the ability to achieve micron-level precise drive and self-perception.

## 5. Other Smart Materials

In addition to the three smart materials mentioned above, there are many other materials that play an important role in the aerospace industry that are worth mentioning. For example, magnetorheological material is an advanced material that disperses magnetic substances into the base carrier fluid. The volume fraction of ferromagnetic particles in magnetorheological materials is usually between 20~40%, and its rheological properties can be controlled by the applied magnetic field. When the magnetic field strength is different, the performance of magnetorheological materials is also different, and this characteristic of magnetorheological materials is called the magnetorheological effect. The main application directions of magnetorheological materials in the aerospace industry are: improving engine propulsion performance, aircraft head heat control, nozzle thrust vector control, replacing traditional power generation devices, etc. [149]. For a hypersonic vehicle that use ramjet as power because the engine does not have rotating parts, it is impossible to use a traditional method of discharge, and independent or embedded magnetic–fluid power generation can be used to provide power for the long time of the on-board equipment of the aircraft. Considering the overall design, high power, thrust loss, and volume weight, the battery plan is difficult to meet the requirements; magnetic–fluid power generation may be the most feasible project and deserves attention and research. Magnetorheological control has a good application prospect for adjusting or improving the performance of the intake airway, and the use of magnetic field excitation can expand its Mach number usage range.

For a variety of reasons, magneto-electro-elastic (MEE) materials are superior to general smart materials. A large part of this is attributed to the multifunctional behavior and physical properties of these materials. In addition to energy conversion and harvesting, MEE composites also have self-sensing and self-actuation functions [150]. MEE sensors are coming into view where a couple field behavior sensors are expected to sense multiple loads of different kinds [151]. Arunkumar et al. [152] analyzed the vibroacoustic response of sandwich panel structures, such as honeycomb, trapezoidal, and triangular, which are widely used in aerospace structures, and provided a precise solution for the vibroacoustic response of MEE, which is of great significance for the further application and research of MEE materials in the aerospace industry.

Additive manufacturing (AM) technology is a technology that relies on special manufacturing processes to produce high-quality materials, and auxiliary structure is a specially designed composite structure; the use of additive technology to manufacture auxiliary structures is a hot research direction. The vacuum resin infusion technology mentioned above is a class of auxiliary structure manufacturing technology, and there are many such applications in the aviation industry. Macroscopic and microscopic auxetic structures are used in aerospace components, such as curved body parts, aircraft nose-cones, and aircraft wing panels [153]. In addition, auxiliary structures have also been studied in the direction of active cooling of supersonic vehicles [154] and shock absorption [155]. Joseph et al. [156] provided an interesting review of the literature on the application of auxiliary structures.

## 6. Conclusions

In this paper, we systematically summarize and report the existing or possible application directions of smart materials represented by piezoelectric materials, shape memory materials, giant magnetostrictive materials, and some other materials in the aerospace industry, briefly describing the basic implementation schemes of different smart structures and application directions, introducing the advantages, disadvantages, and development status of different smart materials in different smart structure applications, and having an enlightened effect on the development of related research.

In conclusion, piezoelectric materials have the most extensive and deep applications; shape memory materials can reconcile the contradiction between different design purposes on shape requirements; and giant magnetostrictive materials have a unique high-precision actuation mode, which is expected to have deeper applications in the design of aircraft actuators in the future. The overall application trend of smart materials is to obtain higher safety, a longer life cycle, higher robustness, and stronger controllability, and its control strategy is to develop from a single and linear control mode to a composite and nonlinear control direction; the function of the smart structure itself tends to achieve higher resolution, obtain a wider coverage bandwidth, and realize the integration of sensing and actuation.

Piezoelectric materials, shape memory materials, and giant magnetostrictive materials, as the three most representative intelligent materials, have in-depth research and development in the aerospace industry and other fields. The intelligent structure functions cover various fields in the aviation industry, such as structural health monitoring, energy harvesting, vibration control, high-precision displacement output, shape control, etc.

In general, the function of intelligent structures tends to give priority to solving the safety problem under complex aerospace application conditions. Under the guidance of this goal, the research scope of structural health monitoring is expanded to the whole life cycle of intelligent structures from preparation to service. Piezoelectric materials play a huge role in the direction of structural health monitoring and SMART layer technology, and Lamb wave nondestructive testing technology is its foundation. 

Although not as in-depth as the application research of piezoelectric materials, shape memory materials and giant magnetostrictive materials also have a development momentum in structural health monitoring, among which giant magnetostrictive materials show better guided wave excitation ability in structural health monitoring applications, and subsequent development can focus on the design of a complete structural health monitoring system using giant magnetostrictive materials and verify it theoretically and practically. 

As an energy supply solution for sensors and actuators, energy harvesting technology should strengthen the research on different energy source collection technologies, improve the efficiency of energy collection, and establish a sound multi-field coupling theoretical model. 

Vibration control not only has performance requirements for the smart material itself but also has requirements for the control strategy. In addition to studying and preparing smart materials with better performance, it is also necessary to further optimize the control strategy to achieve the development from linear control to nonlinear control so as to obtain higher robustness and be combined to cover a wider frequency range. 

As a technology to reconcile different design goals and functional contradictions, structural deformation technology can study the replacement of traditional structures with intelligent actuated structures with better performance and more advanced structures, and further improve the reliability of deformation systems. Another idea is to explore the direct deformation of the structure, abandon the traditional actuated structure, realize the overall continuity of the deformed structure, study the controllability and observability of the overall deformation behavior in depth, and further improve the deformation limit within the scope of application. 

The high-precision displacement output capability of giant magnetostrictive materials is its unique advantage, which has great potential in future aerospace applications, and achieving higher precision displacement output and micro-vibration control applications is of great value for the improvement of interstellar exploration and other capabilities.

## Data Availability

The data presented in this study are available on request from the corresponding author.

## References

[1] Basheer A. (2020). Advances in the smart materials applications in the aerospace industries. Aircr. Eng. Aerosp. Technol..

[2] Aabid A., Parveez B., Raheman M.A., Ibrahim Y.E., Anjum A., Hrairi M., Parveen N., Zayan J.M. (2021). A Review of Piezoelectric Material-Based Structural Control and Health Monitoring Techniques for Engineering Structures: Challenges and Opportunities. Actuators.

[3] Güemes A., Fernandez-Lopez A., Pozo A.R., Sierra-Pérez J. (2020). Structural Health Monitoring for Advanced Composite Structures: A Review. J. Compos. Sci..

[4] Salmanpour M.S., Khodaei Z.S., Aliabadi M.H.F. (2017). Impact Damage Localisation with Piezoelectric Sensors under Operational and Environmental Conditions. Sensors.

[5] Ebrahimi F., Dabbagh A. (2019). Wave Propagation Analysis of Smart Nanostructures.

[6] Rocha H., Semprimoschnig C., Nunes J.P. (2021). Sensors for process and structural health monitoring of aerospace composites: A review. Eng. Struct..

[7] He J.Z., Yuan F.G. (2016). Lamb wave-based subwavelength damage imaging using the DORT-MUSIC technique in metallic plates. Struct. Health Monit..

[8] Wang Y., Qiu L., Luo Y.J., Ding R., Jiang F. (2020). A piezoelectric sensor network with shared signal transmission wires for structural health monitoring of aircraft smart skin. Mech. Syst. Signal Proc..

[9] Wang Y., Qiu L., Luo Y.J., Ding R. (2021). A stretchable and large-scale guided wave sensor network for aircraft smart skin of structural health monitoring. Struct. Health Monit..

[10] Hoshyarmanesh H., Abbasi A. (2018). Structural health monitoring of rotary aerospace structures based on electromechanical impedance of integrated piezoelectric transducers. J. Intell. Mater. Syst. Struct..

[11] Lin M., Chang F.K. (2002). The manufacture of composite structures with a built-in network of piezoceramics. Compos. Sci. Technol..

[12] Qing X.L.P., Beard S.J., Kumar A., Chan H.L., Ikegami R. (2006). Advances in the development of built-in diagnostic system for filament wound composite structures. Compos. Sci. Technol..

[13] Qing X.P., Beard S.J., Ikegami R., Chang F.-K., Boller C. (2009). Aerospace Applications of SMART Layer Technology. Encyclopedia of Structural Health Monitoring.

[14] Qing X.P., Wang Y., Gao L., Kumar A. Distributed multifunctional sensor network for composite structural state sensing. Proceedings of the Sensors and Smart Structures Technologies for Civil, Mechanical, and Aerospace Systems 2012.

[15] Yu Y.H., Liu X., Li J., Wang Y.S., Qing X.L. (2022). Life-cycle health monitoring of composite structures using piezoelectric sensor network. Smart Mater. Struct..

[16] Zhang T., Biswal S., Wang Y. (2020). SHMnet: Condition assessment of bolted connection with beyond human-level performance. Struct. Health Monit..

[17] Konstantopoulos S., Fauster E., Schledjewski R. (2014). Monitoring the production of FRP composites: A review of in-line sensing methods. Express Polym. Lett..

[18] Qing X.L., Liu X., Zhu J.J., Wang Y.S. (2021). In-situ monitoring of liquid composite molding process using piezoelectric sensor network. Struct. Health Monit..

[19] Liu X., Li Y.K., Zhu J.J., Wang Y.S., Qing X.L. (2021). Monitoring of resin flow front and degree of cure in vacuum-assisted resin infusion process using multifunctional piezoelectric sensor network. Polym. Compos..

[20] Ostachowicz W., Soman R., Malinowski P. (2019). Optimization of sensor placement for structural health monitoring: A review. Struct. Health Monit..

[21] Elahi H., Munir K., Eugeni M., Abrar M., Khan A., Arshad A., Gaudenzi P. (2020). A Review on Applications of Piezoelectric Materials in Aerospace Industry. Integr. Ferroelectr..

[22] Das Mahapatra S., Mohapatra P.C., Aria A.I., Christie G., Mishra Y.K., Hofmann S., Thakur V.K. (2021). Piezoelectric Materials for Energy Harvesting and Sensing Applications: Roadmap for Future Smart Materials. Adv. Sci..

[23] Kaur N., Mahesh D., Singamsetty S. (2020). An experimental study on piezoelectric energy harvesting from wind and ambient structural vibrations for wireless structural health monitoring. Adv. Struct. Eng..

[24] Arnold D.P. (2007). Review of microscale magnetic power generation. IEEE Trans. Magn..

[25] Bouma A., Le E., Vasconcellos R., Abdelke A. (2022). Effective design and characterization of flutter-based piezoelectric energy harvesters with discontinuous nonlinearities. Energy.

[26] Cao D.X., Wang J.R., Guo X.Y., Lai S.K., Shen Y.J. (2022). Recent advancement of flow-induced piezoelectric vibration energy harvesting techniques: Principles, structures, and nonlinear designs. Appl. Math. Mech..

[27] Liu Q., Xu Y., Kurths J., Liu X.C. (2022). Complex nonlinear dynamics and vibration suppression of conceptual airfoil models: A state-of-the-art overview. Chaos.

[28] Beran P.S., Strganac T.W., Kim K., Nichkawde C. (2004). Studies of store-induced limit-cycle oscillations using a model with full system nonlinearities. Nonlinear Dyn..

[29] Bao C.Y., Dai Y.T., Wang P., Tang G.J. (2019). A piezoelectric energy harvesting scheme based on stall flutter of airfoil section. Eur. J. Mech. B-Fluids.

[30] Rong Z., Cao B.C., Hu J.Q. (2017). Stability analysis on an aeroelastic system for design of a flutter energy harvester. Aerosp. Sci. Technol..

[31] Bouma A., Vasconcellos R., Abdelkefi A. (2022). Investigations on the stability and effectiveness of wing-based piezoaeroelastic systems with combined nonlinearities. Eur. Phys. J.-Spec. Top..

[32] Pusty M., Shirage P.M. (2022). Insights and perspectives on graphene-PVDF based nanocomposite materials for harvesting mechanical energy. J. Alloys Compd..

[33] Martins P., Lopes A.C., Lanceros-Mendez S. (2014). Electroactive phases of poly(vinylidene fluoride): Determination, processing and applications. Prog. Polym. Sci..

[34] Gu L., Liu J.M., Cui N.Y., Xu Q., Du T., Zhang L., Wang Z., Long C.B., Qin Y. (2020). Enhancing the current density of a piezoelectric nanogenerator using a three-dimensional intercalation electrode. Nat. Commun..

[35] Tommasino D., Moro F., Bernay B., Woodyear T.D., Corona E.D., Doria A. (2022). Vibration Energy Harvesting by Means of Piezoelectric Patches: Application to Aircrafts. Sensors.

[36] Covaci C., Gontean A. (2020). Piezoelectric Energy Harvesting Solutions: A Review. Sensors.

[37] Sheeraz M.A., Malik M.S., Rahman K., Elahi H., Khurram M., Eugeni M., Gaudenzi P. (2022). Multimodal piezoelectric wind energy harvester for aerospace applications. Int. J. Energy Res..

[38] Elahi H. (2021). The investigation on structural health monitoring of aerospace structures via piezoelectric aeroelastic energy harvesting. Microsyst. Technol..

[39] Liu J.L., Bao B., Chen J.T., Wu Y.F., Wang Q. (2023). Passively adaptive wind energy harvester featuring a double-airfoil bluff body with adjustable attack angles. Mech. Syst. Signal Proc..

[40] Li J.J., Li S., He X.F., Yang X.K., Ye Y.Z., Li J.H. (2022). Geometrically nonlinear model of piezoelectric wind energy harvesters based on vortex-induced vibration and galloping. Smart Mater. Struct..

[41] Zhou L.W., Chen G.P. (2017). Fuzzy sliding mode control of flexible spinning beam using a wireless piezoelectric stack actuator. Appl. Acoust..

[42] Rodriguez J., Collet M., Chesne S. (2022). Active Vibration Control on a Smart Composite Structure Using Modal-Shaped Sliding Mode Control. J. Vib. Acoust..

[43] Casagrande D., Gardonio P., Zilletti M. (2017). Smart panel with time-varying shunted piezoelectric patch absorbers for broadband vibration control. J. Sound Vib..

[44] Giraldo G.D., Silva E.C.N., Montealegre R.W. (2020). Topology optimization of piezoelectric sensor and actuator layers for active vibration control. Smart Mater. Struct..

[45] Shtessel Y., Edwards C., Fridman L., Levant A. (2014). Sliding Mode Control and Observation.

[46] Qinglei H., Friswell M.I. (2008). Adaptive Sliding Mode Attitude and Vibration Control of Flexible Spacecraft Under Unknown Disturbance and Uncertainty. IFAC Proc. Vol..

[47] Sharma S., Kumar A., Kumar R., Talha M., Vaish R. (2020). Active vibration control of smart structure using poling tuned piezoelectric material. J. Intell. Mater. Syst. Struct..

[48] Williams D., Khodaparast H.H., Jiffri S., Yang C. (2019). Active vibration control using piezoelectric actuators employing practical components. J. Vib. Control.

[49] Luo H., Wu X., Liu G., Fu J. (2022). Active vibration suppression of intelligent piezoelectric material MFC on solar array panel. J. Vib. Eng..

[50] Kundu A., Berry A. (2011). Active sound control with smart foams using piezoelectric sensoriactuator. J. Intell. Mater. Syst. Struct..

[51] Akl W.N., Baz A. (2007). Finite element Modeling of smart foam for active vibration and noise control applications. Mech. Adv. Mater. Struct..

[52] Wu C.M., Chou M.H. (2016). Polymorphism, piezoelectricity and sound absorption of electrospun PVDF membranes with and without carbon nanotubes. Compos. Sci. Technol..

[53] Kim B.S., Kwon S., Jeong S., Park J. (2019). Semi-active control of smart porous structure for sound absorption enhancement. J. Intell. Mater. Syst. Struct..

[54] Talebitooti R., Gohari H.D., Zarastvand M., Loghmani A. (2019). A robust optimum controller for suppressing radiated sound from an intelligent cylinder based on sliding mode method considering piezoelectric uncertainties. J. Intell. Mater. Syst. Struct..

[55] Wu N. (2012). Structural Repair using Smart Materials. J. Aeronaut. Aerosp. Eng..

[56] Wang Q., Quek S., Liew K. (2002). On the repair of a cracked beam with a piezoelectric patch. Smart Mater. Struct..

[57] Wang Q., Quek S.T. (2004). Repair of delaminated beams via piezoelectric patches. Smart Mater. Struct..

[58] Wang Q., Duan W.H., Quek S.T. (2004). Repair of notched beam under dynamic load using piezoelectric patch. Int. J. Mech. Sci..

[59] Liu T.J.C. (2007). Fracture mechanics and crack contact analyses of the active repair of multi-layered piezoelectric patches bonded on cracked structures. Theor. Appl. Fract. Mech..

[60] Newman J.C., Raju I.S. (1981). An empirical stress-intensity factor equation for the surface crack. Eng. Fract. Mech..

[61] Abuzaid A., Hrairi M., Dawood M. (2018). Evaluating the Reduction of Stress Intensity Factor in Center-Cracked Plates Using Piezoelectric Actuators. Actuators.

[62] Abuzaid A., Hrairi M., Dawood M. (2018). Experimental and numerical analysis of piezoelectric active repair of edge-cracked plate. J. Intell. Mater. Syst. Struct..

[63] Abuzaid A., Hrairi M., Dawood M. (2017). Modeling approach to evaluating reduction in stress intensity factor in center-cracked plate with piezoelectric actuator patches. J. Intell. Mater. Syst. Struct..

[64] Kumar R., Pathak H., Singh A., Tiwari M. (2021). Modeling of crack repair using piezoelectric material: XFEM approach. Eng. Comput..

[65] Sohn H., Park G., Wait J.R., Limback N.P., Farrar C.R. (2004). Wavelet-based active sensing for delamination detection in composite structures. Smart Mater. Struct..

[66] Hameed M.S., Li Z., Zheng K.H. (2020). Damage Detection Method Based on Continuous Wavelet Transformation of Lamb Wave Signals. Appl. Sci..

[67] Hong X.B., Liu Y., Lin X.H., Luo Z.Q., He Z.W. (2018). Nonlinear Ultrasonic Detection Method for Delamination Damage of Lined Anti-Corrosion Pipes Using PZT Transducers. Appl. Sci..

[68] Kuriakose V.M., Sreehari V.M. (2021). Experimental investigation on the enhancement of vibration and flutter characteristics of damaged composite plates using piezoelectric patches. Compos. Struct..

[69] Tabrizikahou A., Kuczma M., Lasecka-Plura M., Farsangi E.N., Noori M., Gardoni P., Li S.F. (2022). Application and modelling of Shape-Memory Alloys for structural vibration control: State-of-the-art review. Constr. Build. Mater..

[70] Matsui R., Takeda K., Tobushi H., Pieczyska E.A. (2018). Mechanical properties and advanced subjects in shape memory alloys and polymers. J. Theor. Appl. Mech..

[71] Pitt D.M., Dunne J.P., White E.V. SAMPSON smart inlet design overview and wind tunnel test: II. Wind tunnel test. Proceedings of the Smart Structures and Materials 2002: Industrial and Commercial Applications of Smart Structures Technologies.

[72] Pitt D., Dunne J., White E., Garcia E. SAMPSON smart inlet SMA powered adaptive lip design and static test. Proceedings of the 19th AIAA Applied Aerodynamics Conference.

[73] Arbogast D., Ruggeri R., Bussom R. Development of a ¼-scale NiTinol actuator for reconfigurable structures. Proceedings of the Industrial and Commercial Applications of Smart Structures Technologies.

[74] Calkins F.T., Mabe J.H. (2010). Shape Memory Alloy Based Morphing Aerostructures. J. Mech. Des..

[75] Mabe J., Gravatt L., Bushnell G., Gutmark E., DiMicco R., Harris C. Shape Memory Alloy Actuators for Deployable Rotor Blade Aerodynamic Devices. Proceedings of the 46th AIAA Aerospace Sciences Meeting and Exhibit 2008.

[76] Hodgson D., Russell S. (2000). Nitinol melting, manufacture and fabrication. Minim. Invasive Ther. Allied Technol..

[77] Calkins T., Butler G., Mabe J. Variable Geometry Chevrons for Jet Noise Reduction. Proceedings of the 12th AIAA/CEAS Aeroacoustics Conference (27th AIAA Aeroacoustics Conference).

[78] Jani J.M., Leary M., Subic A., Gibson M.A. (2014). A review of shape memory alloy research, applications and opportunities. Mater. Des..

[79] Costanza G., Tata M.E. (2020). Shape Memory Alloys for Aerospace, Recent Developments, and New Applications: A Short Review. Materials.

[80] Ajaj R.M., Parancheerivilakkathil M.S., Amoozgar M., Friswell M.I., Cantwell W.J. (2021). Recent developments in the aeroelasticity of morphing aircraft. Prog. Aerosp. Sci..

[81] Hao L., Qiu J.H., Ji H.L., Nie R. (2018). Numerical analysis on shape memory alloy-based adaptive shock control bump. J. Intell. Mater. Syst. Struct..

[82] Qiu J.H., Hao L., Ji H.L., Zhang C., Nie R. (2022). Use of shape memory alloy for active shock control bump application. J. Intell. Mater. Syst. Struct..

[83] Kunnecke S.C., Vasista S., Riemenschneider J., Keimer R., Kintscher M. (2021). Review of Adaptive Shock Control Systems. Appl. Sci..

[84] Balzarek C., Kalow S., Riemenschneider J., Rivero A. (2022). Manufacturing and Testing of a Variable Chord Extension for Helicopter Rotor Blades. Actuators.

[85] Ameduri S., Ciminello M., Concilio A., Dimino I., Galasso B., Guida M., Miceli M.F., Riemenschneider J., Kalow S., Luebker J. (2022). Whirl Tower Demonstration of an SMA Blade Twist System. Actuators.

[86] Botez R.M., Popov A.V., Courchesne S. New aeroelastic studies for a morphing wing. Proceedings of the 48th AIAA Aerospace Sciences Meeting including the New Horizons Forum and Aerospace Exposition.

[87] Ashir M., Hindahl J., Nocke A., Cherif C. (2020). Development of an adaptive morphing wing based on fiber-reinforced plastics and shape memory alloys. J. Ind. Text..

[88] Hajarian A., Zakerzadeh M.R., Baghani M. (2019). Design, analysis and testing of a smart morphing airfoil actuated by SMA wires. Smart Mater. Struct..

[89] Godard O.J., Lagoudas M.Z., Lagoudas D.C. Design of space systems using shape memory alloys. Proceedings of the Smart Structures and Materials 2003: Smart Structures and Integrated Systems.

[90] Yan X.J., Huang D.W., Zhang X.Y., Liu Y., Yang Q.L. (2015). A one-stage, high-load capacity separation actuator using anti-friction rollers and redundant shape memory alloy wires. Rev. Sci. Instrum..

[91] Huang D.W., Yan X.J., Zhang X.Y., Bai H.B., Wang X., Liu Y. (2017). A SMA wire actuated extremely long-lifetime release actuator using two ball-lock mechanisms. Rev. Sci. Instrum..

[92] Yang F., Yue H.H., Zhang Y.L., Peng J.S., Deng Z.Q. (2017). Research on a low-impact unlocking trigger device of heavy load based on shape memory alloy fiber. Adv. Mech. Eng..

[93] Li F.F., Liu L.W., Lan X., Pan C.T., Liu Y.J., Leng J.S., Xie Q. (2019). Ground and geostationary orbital qualification of a sunlight-stimulated substrate based on shape memory polymer composite. Smart Mater. Struct..

[94] Lan X., Liu L.W., Pan C.T., Li F.F., Liu Z.X., Hou G.H., Sun J., Dai W.X., Wang L.L., Yue H.H. (2021). Smart Solar Array Consisting of Shape-Memory Releasing Mechanisms and Deployable Hinges. AIAA J..

[95] Lan X., Liu L.W., Zhang F.H., Liu Z.X., Wang L.N., Li Q.F., Peng F., Hao S.D., Dai W.X., Wan X. (2020). World’s first spaceflight on-orbit demonstration of a flexible solar array system based on shape memory polymer composites. Sci. China-Technol. Sci..

[96] Qiu J., Stiharu I. (2022). Vibration and noise reduction of pipelines using shape memory alloy. Sci. Eng. Compos. Mater..

[97] Bidaux J.E., Bernet N., Sarwa C., Manson J.A.E., Gotthardt R. (1995). Vibration frequency control of a polymer beam using embedded shape-memory-alloy fibres. J. Phys..

[98] Garafolo N.G., McHugh G.R. (2018). Mitigation of flutter vibration using embedded shape memory alloys. J. Fluids Struct..

[99] Mani Y., Veeraragu J., Sangameshwar S., Rangaswamy R. (2020). Dynamic behavior of smart material embedded wind turbine blade under actuated condition. Wind Struct..

[100] Kuo S.Y., Shiau L.C., Lai C.H. (2012). Flutter of buckled shape memory alloy reinforced laminates. Smart Mater. Struct..

[101] Lin H.G., Shao C.H., Cao D.Q. (2020). Nonlinear flutter and random response of composite panel embedded in shape memory alloy in thermal-aero-acoustic coupled field. Aerosp. Sci. Technol..

[102] Baitab D.M., Majid D.L., Abdullah E.J., Hamid M.F.A., Jang L.S., Haider I.M. (2022). Flutter performance of shape memory alloy-embedded 3D woven flexible composite plate under subsonic flow. J. Ind. Text..

[103] Bhaskar J., Kumar S.A., Bhattacharya B., Adhikari S. (2020). A review on shape memory alloy reinforced polymer composite materials and structures. Smart Mater. Struct..

[104] Saadat S., Salichs J., Noori M., Hou Z., Davoodi H., Bar-On I., Suzuki Y., Masuda A. (2002). An overview of vibration and seismic applications of NiTi shape memory alloy. Smart Mater. Struct..

[105] Kwon S.C., Jeon S.H., Oh H.U. (2015). Performance evaluation of spaceborne cryocooler micro-vibration isolation system employing pseudoelastic SMA mesh washer. Cryogenics.

[106] Kwon S.C., Jo M.S., Oh H.U. (2017). Experimental Validation of Fly-Wheel Passive Launch and On-Orbit Vibration Isolation System by Using a Superelastic SMA Mesh Washer Isolator. Int. J. Aerosp. Eng..

[107] Jeong H.K., Han J.H., Youn S.H., Lee J. (2014). Frequency tunable vibration and shock isolator using shape memory alloy wire actuator. J. Intell. Mater. Syst. Struct..

[108] Wei Z.G., Sandstrom R., Miyazaki S. (1998). Shape memory materials and hybrid composites for smart systems—Part II Shape-memory hybrid composites. J. Mater. Sci..

[109] Meo M., Antonucci E., Duclaux P., Giordano M. (2005). Finite element simulation of low velocity impact on shape memory alloy composite plates. Compos. Struct..

[110] Gupta A.K., Velmurugan R., Joshi M., Gupta N.K. (2019). Studies on shape memory alloy-embedded GFRP composites for improved post-impact damage strength. Int. J. Crashworthiness.

[111] Padula S.A., Creager C.M. Shape Memory Alloy (SMA) Tires—A New Paradigm in Tire Performance. Proceedings of the Annual Meeting and Conference on Tire Science and Technology (No. GRC-E-DAA-TN59946).

[112] Barik L., Samal S., Behera A., Rajak D.K., Pruncu C.I. (2021). On the replacement of steel by NITINOL as coupling agent in automobile shaft. ISSS J. Micro Smart Syst..

[113] Liu D.Z., Liu W.X., Gong F.Y. (1995). Engineering application of Fe-based shape memory alloy on connecting pipe line. J. Phys..

[114] Jee K.K., Han J.H., Jang W.Y. (2006). A method of pipe joining using shape memory alloys. Mater. Sci. Eng. A.

[115] Cao B., Sun Q., Iwamoto T. (2022). Bending fracture strength of the pipe joint using iron-based shape memory alloy (Fe-SMA) subjected to different expansion methods at various deformation rates. Eng. Struct..

[116] Cerdan K., Van Assche G., van Puyvelde P., Brancart J. (2020). A novel approach for the closure of large damage in self-healing elastomers using magnetic particles. Polymer.

[117] Alabiso W., Hron T.M., Reisinger D., Bautista-Anguis D., Schlogl S. (2021). Shape memory-assisted self-healing of dynamic thiol-acrylate networks. Polym. Chem..

[118] Kuang K.S.C., Cantwell W.J. (2003). The use of plastic optical fibres and shape memory alloys for damage assessment and damping control in composite materials. Meas. Sci. Technol..

[119] Saeedi A., Shokrieh M.M. (2017). Effect of shape memory alloy wires on the enhancement of fracture behavior of epoxy polymer. Polym. Test..

[120] Kirkby E.L., Michaud V.J., Manson J.A.E., Sottos N.R., White S.R. (2009). Performance of self-healing epoxy with microencapsulated healing agent and shape memory alloy wires. Polymer.

[121] Poormir M.A., Khalili S.M.R., Eslami-Farsani R. (2018). Optimal design of a bio-inspired self-healing metal matrix composite reinforced with NiTi shape memory alloy strips. J. Intell. Mater. Syst. Struct..

[122] Saeedi A., Shokrieh M.M. (2019). A novel self-healing composite made of thermally reversible polymer and shape memory alloy reinforcement. J. Intell. Mater. Syst. Struct..

[123] Jony B., Roy S., Mulani S.B. (2020). Fracture resistance of in-situ healed CFRP composite using thermoplastic healants. Mater. Today Commun..

[124] Konlan J., Mensah P., Ibekwe S., Crosby K., Li G.Q. (2020). Vitrimer based composite laminates with shape memory alloy Z-pins for repeated healing of impact induced delamination. Compos. Part B Eng..

[125] Apicella V., Clemente C.S., Davino D., Leone D., Visone C. (2019). Review of Modeling and Control of Magnetostrictive Actuators. Actuators.

[126] Deng Z.X., Dapino M.J. (2018). Review of magnetostrictive materials for structural vibration control. Smart Mater. Struct..

[127] Elhajjar R., Law C.T., Pegoretti A. (2018). Magnetostrictive polymer composites: Recent advances in materials, structures and properties. Prog. Mater. Sci..

[128] Huan Q., Miao H.C., Li F.X. (2018). A variable-frequency structural health monitoring system based on omnidirectional shear horizontal wave piezoelectric transducers. Smart Mater. Struct..

[129] Seung H.M., Kim H.W., Kim Y.Y. (2013). Development of an omni-directional shear-horizontal wave magnetostrictive patch transducer for plates. Ultrasonics.

[130] Zhou L., Yang Y.J., Yuan F.G. (2012). Design of a magnetostrictive sensor for structural health monitoring of non-ferromagnetic plates. J. Vibroeng..

[131] Park C.I., Seung H.M., Kim Y.Y. (2019). Bi-annular shear-horizontal wave MPT tailored to generate the SH1 mode in a plate. Ultrasonics.

[132] Sha G.F., Lissenden C.J. (2021). Modeling Magnetostrictive Transducers for Structural Health Monitoring: Ultrasonic Guided Wave Generation and Reception. Sensors.

[133] Lee J.K., Kim H.W., Kim Y.Y. (2013). Omnidirectional Lamb Waves by Axisymmetrically-Configured Magnetostrictive Patch Transducer. IEEE Trans. Ultrason. Ferroelectr. Freq. Control.

[134] Xie C.X., Liu T.H., Pei C.X., Chen Z.M. (2022). A Flexible Thin-Film Magnetostrictive Patch Guided-Wave Transducer for Structural Health Monitoring. IEEE Sens. J..

[135] Zenkour A.M., El-Shahrany H.D. (2020). Vibration suppression of advanced plates embedded magnetostrictive layers via various theories. J. Mater. Res. Technol..

[136] Yoo J.H., Flatau A.B. (2012). A bending-mode galfenol electric power harvester. J. Intell. Mater. Syst. Struct..

[137] Yan B.P., Zhang C.M., Li L.L. (2015). Design and Fabrication of a High-Efficiency Magnetostrictive Energy Harvester for High-Impact Vibration Systems. IEEE Trans. Magn..

[138] Xu Z.D., Xu F.H., Chen X. (2016). Intelligent Vibration Isolation and Mitigation of a Platform by Using MR and VE Devices. J. Aerosp. Eng..

[139] Sateesh B., Maiti D.K. (2009). Closed-loop active vibration control of a typical nose landing gear with torsional MR fluid based damper. Struct. Eng. Mech..

[140] Chae H.D., Choi S.B. (2015). A new vibration isolation bed stage with magnetorheological dampers for ambulance vehicles. Smart Mater. Struct..

[141] Xu K.F., Zhang Y.W., Zang J., Niu M.Q., Chen L.Q. (2021). Integration of vibration control and energy harvesting for whole-spacecraft: Experiments and theory. Mech. Syst. Signal Proc..

[142] Mirzavand B., Javadi M.G., Atashgah M.A.A. (2023). On Thermally Induced Vibration Control of Hybrid Magnetostrictive Beams and Plates. Int. J. Struct. Stab. Dyn..

[143] Zhu W., Zhang L., Jin X., Deng X., An Z. (2020). Application and Explanation of Giant Magnetostrictive Material in Aircraft. Mech. Res. Appl..

[144] Wang X., Wu H.M., Yang B.T. (2020). Micro-vibration suppressing using electromagnetic absorber and magnetostrictive isolator combined platform. Mech. Syst. Signal Proc..

[145] Sun X.Q., Yang B.T., Hu W., Bai Z. (2021). Simultaneous Precision Positioning and Vibration Control for on-Orbit Optical Payloads: An Integrated Actuator Development and Analysis. J. Vib. Eng. Technol..

[146] Yang Y.Y., Wang L., Tan J.B., Zhao B. (2016). Induced Voltage Linear Extraction Method Using an Active Kelvin Bridge for Disturbing Force Self-Sensing. Sensors.

[147] Apicella V., Clemente C.S., Davino D., Leone D., Visone C. (2019). Self-Sensing Estimation of Mechanical Stress in Magnetostrictive Actuators. IEEE Trans. Magn..

[148] Dongjian X., Yikun Y., Bintang Y. (2022). Self-sensing magnetostrictive actuator based on ΔE effect: Design, theoretical modeling and experiment. Smart Mater. Struct..

[149] Li Y., Zhang B., Li Y., Xiao L., Wang Y., He G. (2017). Applicationgs and prospects of magnetohydrodynamics in aeronautical engineering. Adv. Mech..

[150] Vinyas M. (2021). Computational Analysis of Smart Magneto-Electro-Elastic Materials and Structures: Review and Classification. Arch. Comput. Methods Eng..

[151] Khanmirza E., Jamalpoor A., Kiani A. (2017). Nano-scale mass sensor based on the vibration analysis of a magneto-electro-elastic nanoplate resting on a visco-Pasternak substrate. Eur. Phys. J. Plus.

[152] Arunkumar M.P., Bhagat V., Geng Q., Li Y.M., Pitchaimani J. (2022). An exact solution for vibro-acoustic response of smart sandwich panels with MEE composite Layer. Compos. Struct..

[153] Rana S., Magalhães R.M.P., Fangueiro R. Advanced auxetic fibrous structures and composites for industrial applications. Proceedings of the 7th International Conference on Mechanics and Materials in Design.

[154] Seepersad C.C., Dempsey B.M., Allen J.K., Mistree F., McDowell D.L. (2004). Design of Multifunctional Honeycomb Materials. AIAA J..

[155] Yang L., Harrysson O.L.A., Cormier D., West H.A., Park C.M., Peters K. Design of Auxetic Sandwich Panels for Structural Applications Design of Auxetic Sandwich Panels for Structural Applications. Proceedings of the 2013 International Solid Freeform Fabrication Symposium.

[156] Joseph A., Mahesh V., Harursampath D. (2021). On the application of additive manufacturing methods for auxetic structures: A review. Adv. Manuf..

